# A Novel Design Method for Energy Absorption Property of Chiral Mechanical Metamaterials

**DOI:** 10.3390/ma14185386

**Published:** 2021-09-17

**Authors:** Mengli Ye, Liang Gao, Fuyu Wang, Hao Li

**Affiliations:** 1 The State Key Lab of Digital Manufacturing Equipment and Technology, Huazhong University of Science and Technology, 1037 Luoyu Road, Wuhan 430074, China; yml1991@hust.edu.cn (M.Y.); gaoliang@mail.hust.edu.cn (L.G.); 2 AVIC Shenyang Aircraft Design and Research Institute, 40 Tawan Road, Shenyang 110000, China; wangfuyu601@163.com

**Keywords:** full-cycle interactive progressive method, energy absorption property, rotation mechanism, chiral mechanical metamaterials

## Abstract

In this paper, a full-cycle interactive progressive (FIP) method that integrates topology optimization, parametric optimization, and experimental analysis to determine the optimal energy absorption properties in the design of chiral mechanical metamaterials is proposed. The FIP method has improved ability and efficiency compared with traditional design methods due to strengthening the overall design, introducing surrogate models, and its consideration of the application conditions. Here, the FIP design was applied in the design of mechanical metamaterials with optimized energy absorption properties, and a chiral mechanical metamaterial with good energy absorption and impact resistance was obtained based on the rotation mechanism of metamaterials with a negative Poisson’s ratio. The relationship among the size parameters, applied boundary conditions, and energy absorption properties were studied. An impact compression experiment using a self-made Fiber Bragg Grating sensor was carried out on the chiral mechanical metamaterial. In light of the large deviation of the experimental and simulation data, a feedback adjustment was carried out by adjusting the structural parameters to further improve the mechanical properties of the chiral mechanical metamaterial. Finally, human–computer interaction, self-innovation, and a breakthrough in the design limits of the optimized model were achieved. The results illustrate the effectiveness of the FIP design method in improving the energy absorption properties in the design of chiral mechanical metamaterials.

## 1. Introduction

Chiral mechanical metamaterials ensure energy absorption and impact resistance through a rotation mechanism. The property of energy absorption is an important indicator used to assess the effect of the rotation mechanism. It has become an important factor in modern industry that affects product quality, operation accuracy, and product life [1,2,3]. Therefore, research on theoretical methods and effective ways to improve structural energy absorption and impact resistance has important practical values.

Currently, the most studied and widely used mechanical metamaterials are those with negative Poisson’s ratios, which have energy absorption and impact resistance properties. They demonstrate outstanding performances in elastic modulus adjustment, indentation resistance, and energy absorption [4]. Negative Poisson’s ratio metamaterials with a rotation mechanism are also called chiral metamaterials, which were first proposed by Kelvin and William in 1904 [5]. Under the action of an external load, the rotation mechanism expands the deformation space of the structure and prolongs the gathering time of the materials. These are conducive to the interlocking the deformation and greatly improve the energy absorption performance of the overall structure [6]. The unique structure and mechanical characteristics of chiral metamaterials have been the subject of extensive attention and research. Davood et al. [7] studied the influence of chirality and the number of layers on the response of a honeycomb structure. Their results showed that the chiral configuration reduced the overall mechanical properties, but the multilayer structure improved the stiffness of the hexagonal honeycomb. The chiral structure of four reverse ligaments showed strong anisotropy and swelling and low shear stiffness. Tancogne-Dejean et al. [8] introduced geometric parameters to control the chiral mechanism. Finite element analysis was used to study the mechanical properties, and tensile testing was utilized to verify the simulation results. Bacigalupo et al. [9] investigated the optimal spectral design of lattice materials and metamaterials, and developed tailored hexachiral, tetrachiral, and anti-tetrachiral metamaterials with good dispersion spectra. From the perspective of the band gap theory, here, an effective design method for chiral metamaterials, the FIP method, is explored. At present, most research on chiral structures has been based on the theoretical design of ligament structures. Optimization designs based on mechanical properties, such as energy absorption and impact resistance, are insufficient. Their application structure is simple, and the innovative configuration still needs further exploration. Therefore, it is necessary to conduct related research on the design and improvement of mechanical metamaterials.

The FIP design method is a full-cycle optimization method that seeks the optimal design goal along a gradient and interactively adjusts the design direction. The FIP method consists of three parts: topology optimization, parametric optimization, and experimental analysis. Topology optimization provides an innovative initial structure for the FIP design and a powerful initial solution for designing chiral metamaterials with enhanced energy absorption performance [10,11]. Kim et al. [12] proposed a topology optimization method using microstructure to represent volume elements. This method takes a specific stress–strain curve as the target rather than aiming at the mechanical parameters. Behrou et al. [13] designed periodic microstructures with specific nonlinear constitutive characteristics within a limited strain range. It is highly suitable for soft robots with vibration reduction functions. Parametric optimization based on a surrogate model is the core stage of FIP design. The surrogate model is a data-driven analysis model that can approximately show the implicit relationships among the design variables, objective functions, and constraints. It can effectively reduce the calculation costs, and its calculation results are comparable with those of the original high-precision commercial structural analysis software [14,15]. The surrogate model was introduced to improve the efficiency of parametric optimization [16], which was used by Li et al. [17] and Lin et al. [18] to improve the kriging model and the neural network algorithm respectively. Some numerical examples have been used to illustrate the effectiveness of these methods in parametric optimization based on surrogate models. The response surface method was used by Cho et al. [19] to optimize the parameters of a metal fixture. This method can effectively reduce the weight of the structure while ensuring the dimensional accuracy of the capsule.

Experimental analysis by impact compression is the verification analysis and feedback adjustment process of FIP design. This process is necessary to ensure the effectiveness of the optimization results. Moreover, the chiral metamaterials obtained by parametric optimization can be distinguished by their complex structure and small macroscopic size. Regarding their manufacture, requirements cannot be satisfied when considering traditional processing methods (machining, injection molding, laser cutting, casting, forging, etc.). Additive manufacturing, also known as three-dimensional printing, is a product manufacturing technology that combines computer-aided design, material processing, and forming. Compared with the traditional material removal and assembly mode, additive manufacturing is a “bottom-up” manufacturing method. This makes it possible to manufacture complex and tiny structures that cannot be easily achieved due to the space and size constraints of traditional manufacturing methods [20,21]. Additive manufacturing provides a feasible solution for the production of metamaterials, and relevant scholars have conducted extensive research work. The advanced two-photon three-dimensional printing technology proposed by Lu et al. [22] was used to produce three-dimensional micro-lattice high-entropy alloy composite mechanical metamaterials at the micro-nano scale for the first time. This pioneered a new way of designing and manufacturing structured metal micro-lattice metamaterials with adjustable mechanical properties. Based on the additive manufacturing method, this study realized high-precision manufacturing of the optimized structures. Moreover, the size and shape accuracy could satisfy the requirements of the subsequent experiment.

In this paper, the topology optimization of rotating properties is presented in Section 2. The parametric optimization of the chiral metamaterials is outlined in Section 3. An impact compression experiment using a self-made Fiber Bragg Grating sensor was carried out on the chiral mechanical metamaterial, as described in Section 4. Section 5 summarizes the entire contents of this paper.

## 2. Topology Optimization of the Rotating Properties

To obtain the rotation mechanism in the optimization model of chiral mechanical metamaterials, the couple stress theory was applied to the conventional concave optimization model. In addition to the independent unidirectional degrees of freedom, the microstructure unit cell applying the couple stress theory also has coupled rotational degrees of freedom. When a chiral structure is rotating in equilibrium, the stress tensor is asymmetric. In order to achieve this rotational asymmetry, the microscopic angle of the rotating structure is also required to be equal to the angle of the macroscopic voxel [23,24].

**Figure 1:**
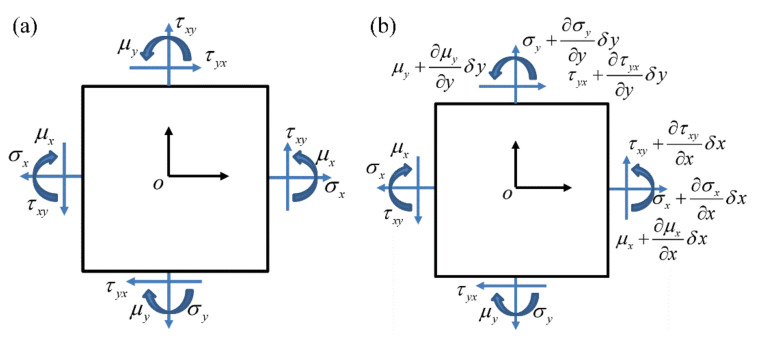
Couple stress analysis of the plane structure. ((**a**) shows the couple stress in the rectangular coordinate system, and (**b**) shows the microstructure unit cell considering the couple stress).

In Figure 1, (a) shows the couple stress in the rectangular coordinate system, and (b) shows the microstructure unit cell considering the couple stress. σ, τ, and μ represent normal stress, shear stress, and couple stress, respectively. According to the force balance equation $\left\{\begin{array}{l} \sum F_{x}=0\\ \sum F_{y}=0 \end{array}\right.$ and the moment balance equation $\sum M_{o}=0$ (o is the centroid of the infinitesimal body) in Figure 1, we obtain

(1) $$\left\{\begin{array}{l} \left(\sigma_{x}+\frac{\partial \sigma_{x}}{\partial x}\delta_{x}-\sigma_{x}\right)\delta_{y}+\left(\tau_{yx}+\frac{\partial \tau_{yx}}{\partial y}\delta_{x}-\tau_{x}\right)\delta_{x}=0\\ \left(\sigma_{y}+\frac{\partial \sigma_{y}}{\partial y}\delta_{y}-\sigma_{y}\right)\delta_{x}+\left(\tau_{xy}+\frac{\partial \tau_{xy}}{\partial x}\delta_{x}-\tau_{xy}\right)\delta_{y}=0 \end{array}\right.$$

(2) $$\begin{array}{l} \left(\mu_{x}+\frac{\partial \mu_{x}}{\partial x}\delta_{x}-m_{x}\right)\delta_{y}+\left(\mu_{y}+\frac{\partial \mu_{y}}{\partial y}\delta_{y}-m_{y}\right)\delta_{x}+\\ \left(\tau_{xy}+\frac{\partial \tau_{xy}}{\partial x}\delta_{x}+\tau_{xy}\right)\delta_{y}\frac{\delta_{x}}{2}-\left(\tau_{yx}+\frac{\partial \tau_{yx}}{\partial y}\delta_{y}+\tau_{yx}\right)\delta_{x}\frac{\delta_{y}}{2}=0 \end{array}$$

From the above stress–strain relationship, the matrix form of the constitutive equation can be obtained as:

(3) $${\left[\sigma ,\mu \right]}^{T}=\left[\begin{array}{cc} C & D\\ D^{T} & B \end{array}\right]{\left[\varepsilon ,\chi \right]}^{T}$$

where σ and μ are the stress and couple stress vectors, respectively; ε and χ are the strain and curvature vectors, respectively; and C and B are the stiffness matrix and the curvature stiffness matrix of the couple stress continuum, respectively. The coupling matrix D assembles the material stress caused by the unit curvature and also indicates the chirality of the material. For achiral materials, the coupling matrix D will disappear [25,26]. The model needs to be solved and analyzed by using the Newton–Raphson iterative method, which is the most widely applicable method used for solving nonlinear finite element equations [27,28,29]. 

In order to design chiral metamaterials, the components of D extracted from the homogenization process should be designed through topology optimization [30,31,32]. In this section, an optimization model of chiral mechanical metamaterials is established based on the homogenization method. The design variable is the expansion coefficient **α** of the level set function during the interpolation by the compactly supported radial basis function. The optimization goal is to maximize $D_{ijkl}$ in the coupling matrix D. The constraint is the volume fraction of the physical material. The specific optimization equation is as follows:

(4) $$\begin{array}{l} \mathrm{find}\;\;\;\;\;\;\alpha =[\alpha_{1},\alpha_{2},\cdots ,\alpha_{N}]\\ \min\;\;\;\;\;\;J=f(D_{ijkl}^{H})\\ s.t.\;\;\;\;\;\;\;\;\left\{\begin{array}{l} a\left(\chi ,v,\Phi \right)=l\left(v,\Phi \right)\;\;\;\;\;\;\;\;\forall \;\;v\in \bar{\;U\;}\left(Y\right)\\ V=\int_{D}H(\phi )d\Omega =\left|Y\right|f_{v}\\ G=\int_{\Omega }dV-V_{\max}\leq 0\\ \alpha_{i,\min}\leq \alpha_{i}\leq \alpha_{i,\max} \end{array}\right.\; \end{array}$$

where i represents the level set node; $\eta_{ijkl}$ represents the weighting factor; $\alpha_{i,\min}$ and $\alpha_{i,\max}$ are the upper and lower bounds of the variation range of the design variables, respectively; and $f_{v}$ is the allowable volume fraction of the structural unit cell. The bilinear form of energy and the linear form of the load in the weak form of the elastic equilibrium condition can be expressed as:

(5) $$\begin{array}{l} a\left(\chi ,v,\varphi \right)=\int_{D}\varepsilon_{ij}^{*}(\chi )D_{ijkl}\varepsilon_{kl}^{*}(v)H(\varphi )d\Omega \\ l\left(v,\varphi \right)\;=\int_{D}\varepsilon_{ij}^{0}D_{ijkl}\varepsilon_{kl}^{*}(v)H(\varphi )d\Omega \end{array}$$

The derivatives of the objective function and the constraint conditions with respect to the design variable $\alpha_{i}$ are

(6) $$\frac{\partial D_{ijkl}^{H}}{\partial \alpha_{i}}=\frac{1}{\left|Y\right|}\int_{D}(\varepsilon_{pq}^{0}-\varepsilon_{pq}^{*}(\chi^{ij}))D_{pqrs}(\varepsilon_{rs}^{0}-\varepsilon_{rs}^{*}(\chi^{kl}))\omega_{i}{\left(x\right)}^{T}\delta (\varphi )d\Omega \;\;$$

(7) $$\frac{\partial V}{\partial \alpha_{i}}=\int_{D}\omega_{i}(x)\delta (\varphi )d\Omega$$

where $\omega_{i}(x)$ is the *i*th node value of the compactly supported radial basis function calculated at the sampling point **x**, and δ(ϕ) is the Dirac function. This section uses the optimization criterion method to perform the topology optimization analysis of the above model.

**Figure 2:**
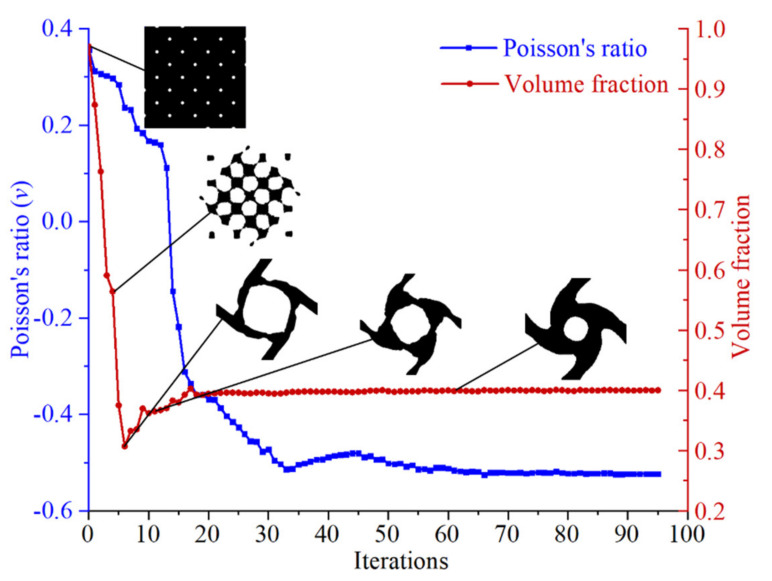
The iterative process of topology optimization of chiral mechanical metamaterials.

The planar four-node quadrilateral elements were used in the nonlinear finite element analysis in the topology optimization process. The volume fraction of the initial design domain is 0.97, and the holes are evenly distributed. One end of the initial design domain is fixed, the opposite end is subjected to unidirectional unit pressure, and the other two sides are free. A new configuration of the chiral mechanical metamaterials can be obtained according to the above optimization model. The diagram in Figure 2 shows the iterative process of topology optimization. Here, we observe that the volume fraction converges to 0.4 after 20 iterations, while the Poisson’s ratio converges to −0.52 after 63 iterations.

**Figure 3:**
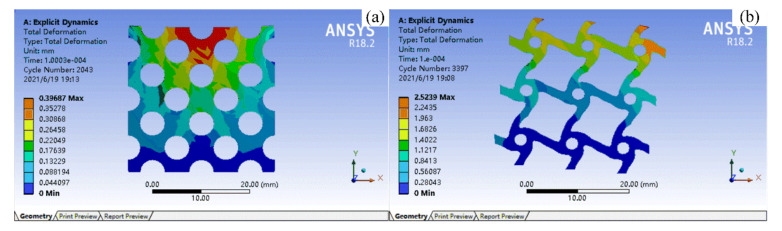
Analysis of the energy absorption properties. ((**a**) shows a uniform porous structure with the same volume fraction as the optimized structure shown in (**b**)).

In practice, the chiral structure is macroscopically presented in the form of a multi-cell structure. In order to verify the energy absorption properties of the chiral structure obtained through topology optimization, a simulation analysis was performed, as shown in Figure 3. Figure 3a shows a uniform porous structure with the same volume fraction as the optimized structure shown in Figure 3b. Through the overall displacement and deformation of the structure, it can be seen that the chiral structure designed through topology optimization has a stronger deformation range and better energy absorption properties with the same volume fraction. Therefore, the optimized structure can be used as the initial configuration for subsequent FIP design to quantify the initial configuration and enhance its energy absorption properties.

## 3. Parametric Optimization of Chiral Metamaterials

### 3.1. Parametric Modeling

Based on the topology optimization, a four-ligament chiral metamaterial unit cell was used as the initial structure for subsequent parameter optimization to enhance its energy absorption properties. In order to conveniently describe the chiral structural characteristics, the shape of the ligament boundary should be simplified when the model is reconstructed in parametric optimization. The fluctuating and disordered structural variables must be reasonably eliminated to ensure the stability and representativeness of the structural parameters of the reconstructed model. The model includes four main aspects: First, the irregular central column of the chiral structure can be simplified into a cylinder. As a supporting connection structure, the cylinder has a better rotation guiding effect that can ensure a smooth connection and avoid local stress concentration during the imposition of force. Secondly, since the unit cell of a chiral structure is centrosymmetric, the parameters of the symmetrical position need to be consistent. Thirdly, the boundaries of the topological configuration are irregular wave curves, which need to be fit into straight lines to extract the characteristic parameters. Lastly, the structures diminutive in size can be ignored, and those in a similar position but larger should be selected.

Uncertainties are inevitable in designing and manufacturing processes, and they will affect the accuracy of the design results eventually. Some scholars have conducted in-depth researches on this issue. The fixed values [33,34] and uncertainties [35] for the material and geometric parameters were used to assess the acoustic performance of the various complex media designs by Sharma et al. Uncertainty in the geometric parameters was observed to have a greater impact compared to the uncertainty in the material properties [35]. Li et al. [36] integrated the Polynomial-Chaos and Chebyshev-Interval functions to perform uncertainty analysis. The material uncertainty on the thermoelastic properties of multiphase composites had been successfully resolved. In this study, materials and loads were deterministic parameters. The geometric uncertainty, i.e., the uncertainty of structural size parameters, was our main consideration. Firstly, uncertainty in parametric optimization was considered by the Kriging model to predict errors. The predicted response obeys a normal distribution-based probability model. Secondly, two data processing methods were adopted in the experiment analysis. (1) The same analyses were performed three times to take the average value as the final result. If there were obvious deviations among these results, the result with the larger deviation should be deleted. The new tests would be added until three groups of similar results were obtained. (2) A series of analysis results of the same factor was fitted based on the MATLAB software by the least square method, and the values with large fluctuations were eliminated. Lastly, the structures from parametric optimization and experiment analysis should be comparatively analyzed and feedback adjustment to reduce the average deviation.

**Figure 4:**
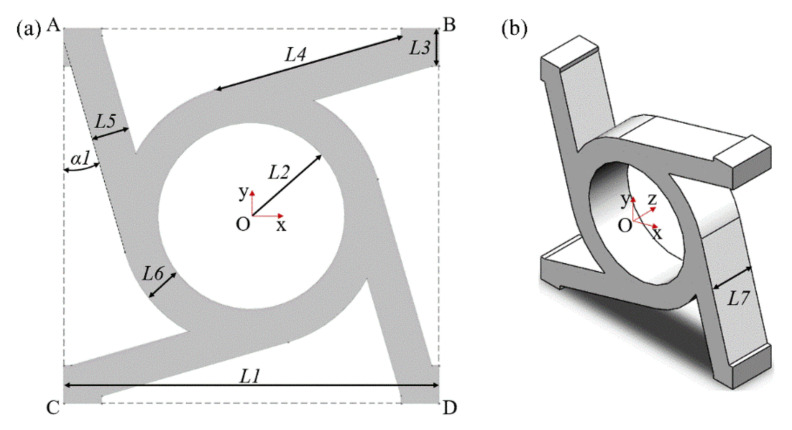
Schematic diagram of the dimensions of the chiral structure. ((**a**) represents the sectional view, and (**b**) represents the stereograph).

Figure 4 shows the schematic diagram of the dimensions of the chiral structure. It can be seen from Figure 4 that the parameterized chiral unit cell includes eight independent size parameters, namely *L1*-*L8* and *α1*. For the convenience of analysis, we set the ligament length *L4* and the width *L5* as being equal. The ligaments are evenly distributed around the axis, and the size of the step at the end of the ligament remains the same. Table 1 shows the parametric value range of the unit cell, which is reasonable under the premise of ensuring the structural integrity and the necessary functions.

**Figure 5:**
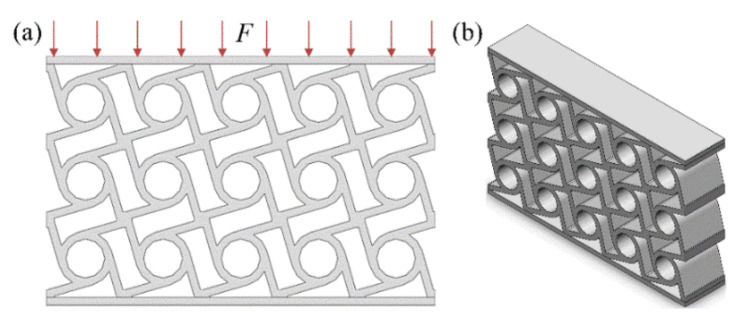
Schematic diagram of the practical chiral structure. ((**a**,**b**) show the schematic cross-sectional view of the practical structure and the three-dimensional view of the practical structure, respectively).

Based on the re-modeled four-ligament chiral structure unit cell in Figure 4, a multi-cell sandwich structure for practical working conditions was constructed, which will be referred to as the practical structure hereafter (Figure 5). Figure 5a,b show the schematic cross-sectional view of the practical structure and the three-dimensional view of the practical structure, respectively. The practical structure includes two main parts. The first part is the upper and lower layers, which are thin plates used to apply external forces and constraints. Adding thin plates at both ends can not only satisfy the packaging requirements of practical applications but also homogenizes the external excitation to avoid stress concentration. The second part is the unit cells of the array. The actual number is determined by the specific usage requirements. For the simulation and experiment process presented here, a structure with 3*5-unit cell array was adopted. Such structures are more in line with actual applications, in which the force status includes the mutual influence of the forces between the cells.

As shown in Figure 5a, F(x) is the external force, and x is the displacement movement of the contact surface in the compression response process of the chiral metamaterial. When the chiral structure is in the densification stage (the solid structure is in full contact in the direction of compression), the corresponding compression surface’ displacement movement is $x_{m}$. This is also the maximum displacement of the chiral structure that can be compressed and deformed. We assumed that the external loss of system energy (i.e., thermal energy and acoustic energy dissipation) during the compression process could be ignored. All the work $W_{out}$ of the external force F(x) is transformed into the energy $E_{in}$ absorbed by the chiral structure. In order to obtain the optimal configuration of chiral metamaterials with optimal energy absorption characteristics, this study took the parameterized structure size as the input variable for the surrogate model. The absorbed energy $E_{in}$ by the chiral structure obtained from the simulation was taken as the output variable of the surrogate model. According to the previous definition, the optimization goal was to obtain the maximum value for energy $E_{in}$ absorbed by the chiral structures. The optimization model is established as follows:

(8) $$\begin{array}{l} \max\;\;\;\;\;E_{in}\\ s.\;\;\;t.\;\;\;\;E_{in}=W_{out}=\int_{0}^{x_{m}}F(x)dx\\ \;\;\;\;\;\;\;\;\;\;\;\;\;\;\;0\leq x\leq x_{m} \end{array}$$

### 3.2. Parametric Optimization Based on a Surrogate Model

The surrogate model used in this paper is based on the effective global optimization of the kriging model. The kriging model is an unbiased estimation model that integrates the regression model and random processes to predict the values of minimum variance and the sample points. As a result, the surrogate model can depict the approximate original model smoothly with only a few sample points and is very suitable for highly non-linear problems [37,38,39]. $X={\left[x_{1},x_{2},\cdot \cdot \cdot ,x_{m}\right]}^{T},(x_{i}\in R^{n})$ and $Y={\left[y_{1},y_{2},\cdot \cdot \cdot ,y_{m}\right]}^{T},(y_{i}\in R^{q})$ are the design variables and response values in the kriging model, respectively. Their relationship can be expressed as follows [40,41]:

(9) Y(X)=g(X)+Z(X)

where g(X) is the deterministic component, and Z(X) is the system deviation. Z(X) is independently and identically distributed and has the following statistical characteristics:

(10) $$\left\{\begin{array}{l} E[Z(X)]=0\\ Var[Z(X)]=\sigma^{2}\\ \mathrm{cov}[Z(x_{i}),Z(x_{j})]=\sigma^{2}R(c,x_{i},x_{j}) \end{array}\right.$$

where $x_{i}$ and $x_{j}$ are arbitrary sample points, $R(c,x_{i},x_{j})$ is a spatial correlation function, and $\sigma^{2}$ is the process variance. The response value Y can be calculated by linear weighted superposition interpolation as follows:

(11) $$\left\{\begin{array}{l} \hat{Y}(X)=w{\left(X\right)}^{T}Y\\ w(X)=R^{-1}(r(X)+G{\left(G^{T}R^{-1}G\right)}^{-1})\cdot (G^{T}R^{-1}r(X)-g(X)) \end{array}\right.$$

where $w(X)=(w_{1},w_{2},\cdot \cdot \cdot ,w_{n})$ are the weight coefficients and $G={\left[g^{T}(x_{1}),g^{T}(x_{2}),\cdot \cdot \cdot ,g^{T}(x_{n})\right]}^{T}$ is a n × k expansion matrix. According to Equation (11), we can obtain the following kriging model:

(12) $$\left\{\begin{array}{l} \hat{Y}(X)=g(X)\beta^{*}+r{\left(X\right)}^{T}R^{-1}(Y-G\beta^{*})\\ R={\left[R_{ij}\right]}_{n\times n}={\left[R(c,x_{i},x_{j})\right]}_{n\times n}\\ r(X)={\left(R(c,x_{1},X),R(c,x_{2},X),\cdot \cdot \cdot ,R(c,x_{n},X)\right)}^{T} \end{array}\right.$$

where $\beta^{*}={\left(G^{T}R^{-1}G\right)}^{-1}G^{T}R^{-1}Y$ is the least-squares estimation of β.

For the parametric optimization of chiral metamaterials based on the surrogate model, first, a reasonable sample space was designed. The sampling points were initially selected as input variables in the sample space using the Latin hypercube sampling method [42]. Each sampling point was distributed as uniformly as possible in the sample space to ensure the global validity of the surrogate model throughout the entire design space. Since the chiral structure had eight independent parameters for the surrogate model analysis, the initial model with D = 8 required 40 sampling points, and the surrogate model needed to calculate 48 sampling points to ensure the accuracy of the optimization results. The sampling criterion was directly related to the speed of convergence and stability, as it was essential for optimization. Here, the effective global optimization algorithm was the sampling criterion adopted to guide updating and optimization of the kriging model in the surrogate model. An optimal response value based on the current design was obtained and points with larger prediction errors could be obtained by maximizing this response value’s expectation [43,44].

**Figure 6:**
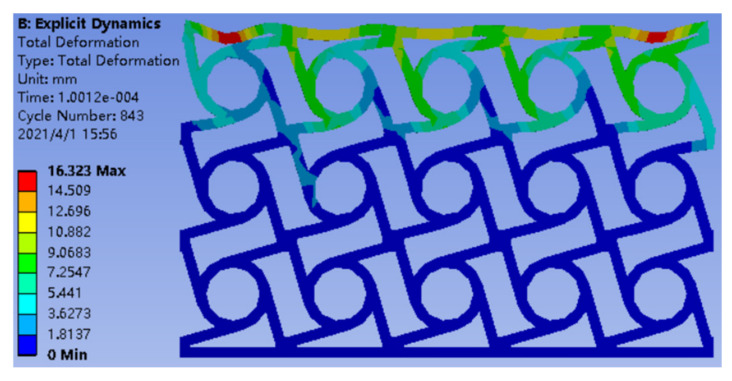
Nephogram of the dynamic simulation of chiral metamaterials.

Secondly, all the simulations and calculations were executed on a computer equipped with an Intel Corei7 CPU with 2.9 GHz and 16 GB of RAM. The ANSYS Workbench 18.2 was used to conduct explicit dynamic analysis. A rotating periodic boundary was created based on a cylindrical coordinate system. Automatic meshing and the grid accuracy were 0.5 mm. There were three main boundary conditions in the finite element simulation and experimental analysis: (1) The external impact force applied to the top of the thin plate was obtained by impacting the thin plate over the same area at a certain speed. Therefore, the external impact force can be regarded as a uniform force in the same direction. (2) The directional impact velocity was 4 × 10^3^ mm/s, and the impact direction is shown in Figure 5. (3) The structure was constrained at the bottom of the thin plate. Table 2 shows the parameters of the chiral structural properties during the simulation analysis. Figure 6 shows a nephogram of the dynamic simulation of chiral metamaterials in the parametric optimization.

Thirdly, a surrogate model containing the mapping relationship between the initial sample points and the output variables was constructed based on adaptive weights and kriging interpolation methods. In the process of adaptive weight control, the maximum fitness value of the spatial samples in the *a*-th iteration was set as $y_{\max}^{a}$. The average fitness value was set as $y_{avg}^{a}=\frac{1}{n}\sum_{i=1}^{n}y_{i}^{a}$. The maximum inertia weight was $w_{\max}=0.9$, and the minimum inertia weight was $w_{\min}=0.4$. If $y_{i}^{a}\geq y_{avg}^{a}$, we obtain $w_{i}^{a}=w_{\min}+(w_{\max}-w_{\min})\cdot \frac{y_{\max}^{a}-y_{i}^{a}}{y_{\max}^{a}-y_{avg}^{a}}$. If $y_{i}^{a}<y_{avg}^{a}$, we obtain $w_{i}^{a}=w_{\max}$.

Finally, the optimization results obtained through the surrogate model were analyzed. If the convergence requirement was not met, the optimization result was added to the sample space to form a new sample set by the effective global optimization criterion. The previous step was repeated to update the surrogate model, and the subsequent optimization design continued. This process cycle was repeated until the optimization process reached the convergence criterion.

### 3.3. Analysis of the Parametric Optimization Results

**Figure 7:**
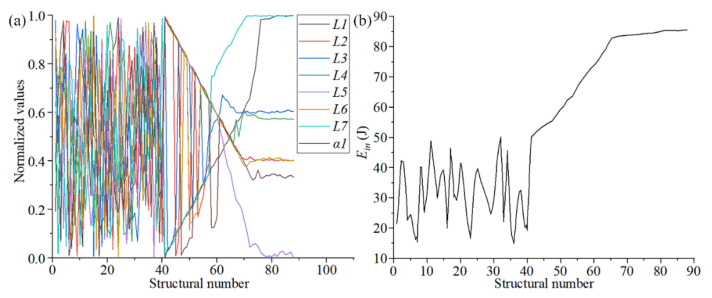
The change trends of the structural parameters and output variables. ((**a**) shows the change trends of the structural parameters during the optimization of chiral metamaterials with the surrogate model. (**b**) shows the change tendency of the output variable P during the optimization process of the surrogate model.).

After completing the parametric optimization of chiral metamaterials, it was necessary to analyze the samples and the corresponding optimization data to evaluate the optimization effect of the surrogate model. Figure 7a shows the change trends of the structural parameters (*L1*, *L2*, *L3*, *L4*, *L5*, *L6*, *L7,* and *α1*) during the optimization of chiral metamaterials with the surrogate model. Owing to the different value ranges of the structural parameters, all the structural parameters in the sample space were normalized to observe the overall change trend under a unified coordinate. It can be observed in Figure 7a that the distribution of structural parameters after convergence was divided into two parts. *L1*, *L5*, and *L7* converge to the boundary value. *L2*, *L3*, *L4*, *L6*, and *α1* converge near the median value. The former are the parameters of the outer framework structure, which mainly controls the unidirectional deformation and overall stiffness of the structure. The latter are the parameters of the internal rotating structure, which mainly controls the rotating linkage deformation of the structure. Figure 7b shows the change tendency of the output variable P during the optimization process of the surrogate model. It can be seen from Figure 7b that the output variables of the optimized structure analyzed through the surrogate model gradually converged to 86.92 J. The overall energy absorption rate increased by more than 63%, which has a strong value for real applications.

**Figure 8:**
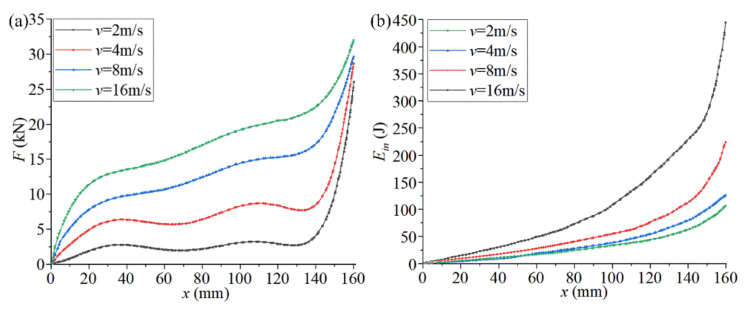
The mechanical characteristic curves of the optimized structures of chiral metamaterials at different impact speeds. ((**a**) shows the compression reaction force-compression displacement curves of the chiral metamaterials at different impact speeds. (**b**) shows the energy absorption curve of chiral metamaterials at different impact speeds).

**Figure 9:**
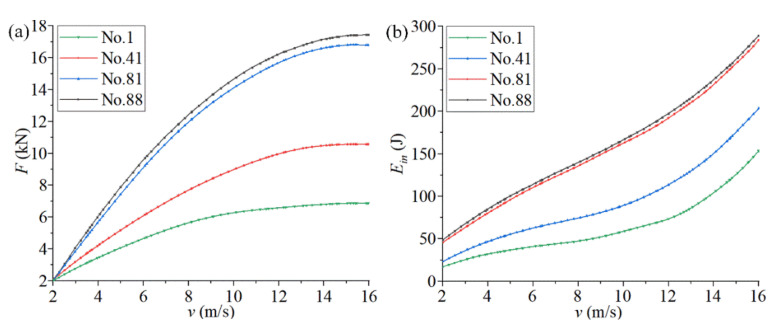
Mechanical properties of chiral metamaterials during parametric optimization. ((**a**,**b**) show the curves of *v*-*F* and *v*-*E_in_*, respectively).

Table 3 shows the optimized structural parameters of the chiral metamaterials. These are the relevant structural parameters indicated by the corresponding sample points after the initial convergence of the surrogate model. In order to observe the superiority of the optimized structure in rotational chirality (also referred to as energy absorption properties) more specifically, Figure 8 and Figure 9 describe the mechanical properties of the chiral metamaterials under different conditions.

The impact velocity in most practical working conditions is less than 16 m/s. Many of the higher speed and more intense collisions involve a sharp deceleration process before the collision contact (the acceleration in this process is very large). For example, for new cars, the frontal 100% overlapping rigid barrier crash test speed has been set to 50 km/h in the Chinese new car assessment program. Figure 8 shows the mechanical characteristic curves of the optimized structures of chiral metamaterials at different impact speeds (2, 4, 8, and 16 m/s). The boundary conditions are given in Figure 5. The size of the contact surface of the rigid cube with the chiral structure during the impact process is guaranteed to be L7*5*L1*, which is fully consistent with the cross-section of the top plate of the chiral structural model. This ensures that the chiral metamaterial is uniformly stressed during the impact process and avoids local deformation and stress concentration, which both affect the overall performance of the structure. 

Figure 8a shows the compression reaction force-compression displacement curves of the chiral metamaterials at different impact speeds. The following can be seen from Figure 8a:

1. The surge state of the initial compression reaction force is the change in stress caused by the impact load on the chiral structure. At this stage, the compression displacement produced by the structural deformation lags behind the compression reaction force, which leads to a sharp increase in the compression reaction force in the initial stage. The final surge state of the compression reaction force is the stage when the walls of solid holes in the chiral structure are in full contact. At this stage, the structural deformation is very small, the external force is close to lossless transmission, and the slight increase in compression displacement causes the compression load to rise sharply. The slow increase in the compression reaction force is the effective stage of the chiral structure. Due to the rotational deformation of the chiral structure, the stress generated by compression of the partial structures will be consumed and transferred. The macroscopic expression is the slow increase in the compression reaction force.
2. As the impact velocity increases, the compression reaction force corresponding to the same compression displacement increases, the compression reaction force leads to increased growth in the slow increase phase, and the compression displacement corresponding to the final surge state of the compression reaction force increases. This is because the greater the impact speed, the lower the efficiency of internal stress consumption and transfer through structural rotation deformation. The macroscopic manifestation is an increase in the compression reaction force and its related state.

Figure 8b shows the energy absorption curve of chiral metamaterials at different impact speeds, which is $E_{in}$ in Equation (8). The following can be seen from Figure 8b:

1. As the compression displacement continues to increase, the energy absorbed by the chiral structure and its acceleration continues to increase. If we compare the energy absorption curves of chiral metamaterials at different impact speeds, it can be found that the energy absorbed by the chiral structure under the same compression displacement increases with an increase in the impact speed. The energy absorption efficiency of the chiral structure is highest in the middle of the compression displacement (80–140 mm).
2. Chiral metamaterials under high-speed impact will enter the nonlinear deformation stage more quickly, and the proportion of this stage is larger. These phenomena show that it is necessary to pre-select the appropriate impact velocity to make full use of the energy absorption properties of chiral structures. The safety interval of the chiral structure can be set, and its utilization efficiency can be improved according to this phenomenon.

In order to evaluate the change law of the mechanical properties of chiral metamaterials in the parametric optimization, we compared the curves of *v*-*F* and *v*-*E_in_* corresponding to different structures (No. 1, No. 41, No. 81, and No. 88) and different speeds (2–16 m/s). Figure 9 shows the mechanical properties of chiral metamaterials during parametric optimization. For the curve of *v*-*F* (Figure 9a), the average compression reaction force in the slow increase stage during impact deformation of the corresponding chiral metamaterial is collected as *F*. For the curve of *v*-*E_in_* (Figure 9b), the absorbed energy corresponding to the end of the force stabilization phase of the compression reaction during the impact deformation process of the corresponding chiral metamaterial is collected as *E_in_*. The following conclusions can be drawn from Figure 9:

1. For the optimized chiral metamaterials, the compression reaction force *F* and the absorbed energy *E_in_* under the same impact velocity were greatly improved. This indicates the effectiveness of parametric optimization for improving the mechanical properties of chiral metamaterials.
2. For any chiral metamaterial structure in the parametric optimization, the compression reaction force *F* and the absorbed energy *E_in_* increase continuously when the impact velocity increases. However, the growth rate of the compression reaction force *F* gradually decreases to zero, and the growth rate of the absorbed energy *E_in_* continues to increase. These indicate that chiral metamaterials have a specific impact velocity range. The energy absorption properties of the chiral metamaterials can be maximized within a suitable range. If the application range is exceeded, the structure will fail, and thus, the energy absorption properties of the chiral metamaterials will be greatly reduced.

## 4. Experimental Analysis of Impact Compression

### 4.1. Specimen Manufacturing and Experimental Design

The chiral metamaterials obtained by the parametric optimization described above have complex structures and a large scale of thin-walled micro-sizes; therefore, traditional manufacturing methods cannot meet the manufacturing requirements. Therefore, in this study, additive manufacturing was chosen for manufacturing the specimens [45,46]. Moreover, the number of sample points in the optimization process of the surrogate model is large, and the experiment would have a high cost and be time-consuming. It is impossible to conduct an experimental analysis on all the sample points used in the surrogate model. In order to save costs and improve the experimental efficiency, we selected some typical structural parameters to manufacture the corresponding test pieces for our experimental analysis. Table 4 provides a list of typical sample specimens used for additive manufacturing. These specimens can completely reflect the structural characteristics of chiral metamaterials at various stages of parametric optimization. The material used for additive manufacturing of the specimens was stainless steel (17–4PH). The size of the manufactured specimens was 15–53 μm, with an elastic modulus of 190 GPa and Poisson’s ratio of 0.305. The AM machine was EOS M290, which includes slicing software. Selective laser melting technology was adopted for additive manufacturing. The selected design software was Magic, and the printing cabin is protected by argon gas.

**Figure 10:**
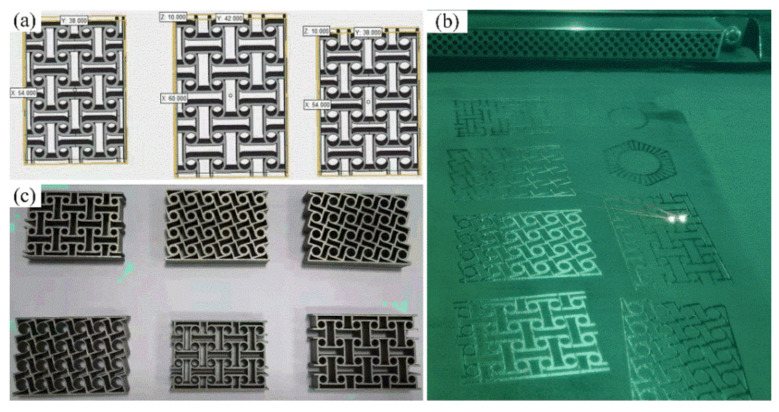
Flow chart of the process for additive manufacturing of the chiral metamaterials. ((**a**) is the basic introduction model, (**b**) is the structure during the forming process, and (**c**) shows some of the final structures).

Figure 10 shows the process for additive manufacturing of the chiral metamaterials, in which (a) is the basic introduction model, (b) is the structure during the forming process, and (c) shows some of the final structures. In the process of additive manufacturing, the typical structures were first introduced into the additive manufacturing software for remodeling. Pre-processing was performed before printing, which includes design optimization, cutting, placement, adding supports, and slicing in accordance with the requirements of additive manufacturing. The additive manufacturing-based primary workpiece can be converted into the final workpiece after post-processing (removal of supports and surface treatment).

In order to analyze the energy absorption properties of the chiral metamaterials obtained above and to verify the effect of parametric optimization for improving the energy absorption properties of the chiral metamaterials, impact experiments were conducted, as described in this section. During the impact compression experiment, the chiral mechanical metamaterials will inevitably experience fatigue when they are subjected to the alternating loads, or the structure repeatedly expands and contracts. However, the focus of this paper is on the maximum allowable energy absorption properties of chiral mechanical metamaterials, which are not related to structural fatigue. In addition, topology optimization and parametric optimization in FIP design are based on the single-objective simulation, which cannot consider the whole process of structural fatigue. Therefore, in order to avoid errors between the results of impact experiments that may have structural fatigue and topology optimization (or parametric optimization) without considering structural fatigue, the following rules should be set for the impact compression experiments. (1) The chiral mechanical metamaterials used in the impact compression experiments are disposable specimens. The workpieces will not be reused even if there is no obvious damage to avoid the influence of structural fatigue. (2) The comparative high speed and great impact energy will be used to ensure that all mechanical parameters are collected in the single-pass deformation without structural spring-back. The impact velocity (2–16 m/s) used in this study can realize the entire process from contact to crushing failure in a single-pass impact compression process. (3) The impact force should be stable and continuous to ensure that the workpiece will not be subjected to alternating loads during the experiments.

**Figure 11:**
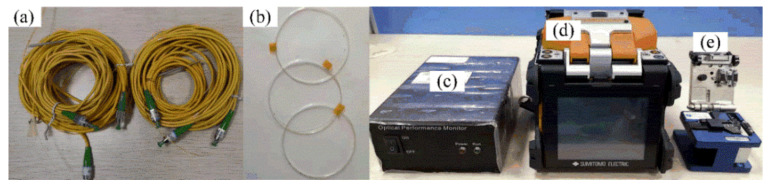
Materials and devices for packaging Fiber Bragg Grating sensors. ((**a**–**e**) show the jumper wires, bare Fiber Bragg Grating, demodulator, fiber welding machine, and fiber cleaver).

**Figure 12:**
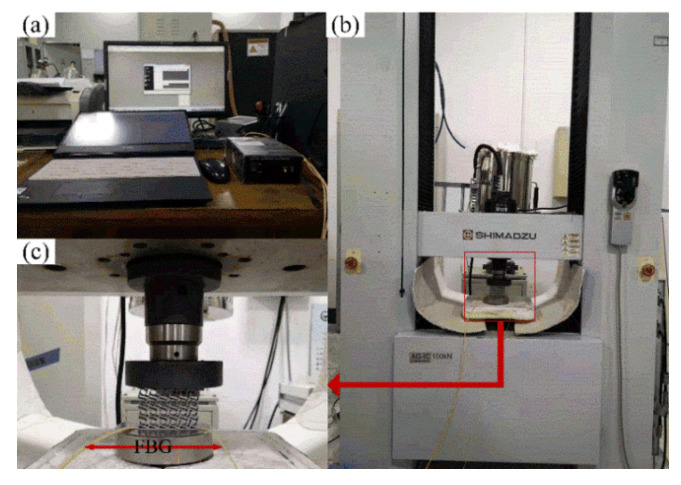
Calibration of the Fiber Bragg Grating pressure sensor. ((**a**) represents the operation interface, and (**c**) is a partly enlarged view of the compression site in (**b**)).

The displacement of specified points, the compression reaction force, the impact velocity, and the output pressure of the chiral metamaterial during the force change process can be detected in impact experiments. The main equipment needed for the impact experiment are high-speed cameras, hydraulic impact testing machines, and Fiber Bragg Grating pressure sensors. The Fiber Bragg Grating pressure sensor needs to be packaged and calibrated before use. This requires a high-temperature durability materials tester and the raw materials of the Fiber Bragg Grating (bare Fiber Bragg Grating, jumper wires, silicone rubber sheet). Figure 11 shows the materials and devices used for packaging the Fiber Bragg Grating pressure sensor. Figure 11a–e show the jumper wires, bare Fiber Bragg Grating, demodulator, fiber welding machine, and fiber cleaver. The sampling frequency selected by the demodulator was 4 kHz, the resolution was 1 pm, and the band was 1525~1565 nm. The Fiber Bragg Grating pressure sensor had to be calibrated before measuring the pressure. It was necessary to obtain the comparison table of the center wavelength drift-pressure through a calibration test. The calibrated Fiber Bragg Grating sensor can reflect the relationship between the pressure and the Fiber Bragg Grating’s center wavelength drift such that the pressure change at a particular location can be monitored in real time. In this section, the AG-IC-100KN-type high-temperature durability material testing machine was used for the calibration. The setup is presented in Figure 12. Figure 12c is a partly enlarged view of the compression site in Figure 12b, including the chiral structure and the Fiber Bragg Grating pressure sensor during calibration.

**Figure 13:**
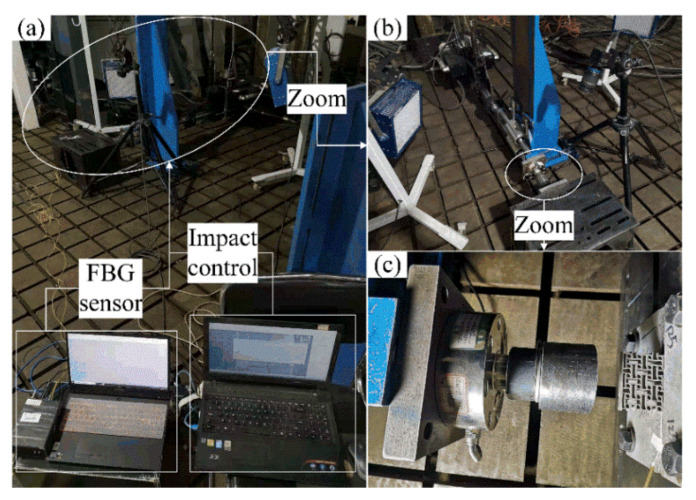
Setup of the specimen compression experiment. ((**a**) is the overall layout of the experimental setup, and (**b**,**c**) are partly enlarged views of the test area and the impact area).

In this study, a microcomputer-controlled impact testing machine was used to carry out the impact load compression experiment in the dynamic response analysis. The setup of the specimen compression experiment is shown in Figure 13, where (a) is the overall layout of the experimental setup, and (b) and (c) are partly enlarged views of the test area and the impact area. In the impact test, the impact head of the hydraulic impact testing machine always compresses the test piece to the maximum displacement before rebounding. The acceleration and pressure sensors on the impact head collect the corresponding data, and the force process of the specimen can be obtained from the subsequent analysis. The high-speed camera captures the entire impact process, and the entire process and characteristics of specimen deformation can be observed from the subsequent analysis. At the same time, the change in displacement of specific positions of the chiral metamaterials can be monitored through the screen. The Fiber Bragg Grating pressure sensor can monitor the change in the output pressure during the impact on the test piece, especially the change in the output pressure after the impact head has separated from the test piece. 

### 4.2. Impact Compression Experiment

The chiral structures obtained by additive manufacturing were used to conduct impact experiments for studying their energy absorption properties under actual conditions. The impact experiment had two main goals: one was to compare and analyze the experimental and simulation data of the chiral structure at different impact speeds. We observed whether they were consistent and proceeded to the subsequent analysis as appropriate. Another goal was to observe whether the mechanical properties of the chiral structure and its changing laws under different impact speeds in the experiment were consistent with the relevant conclusions in the simulation analysis. In order to be consistent with the dynamic simulation analysis in the parametric optimization, the impact load compression experiments conducted here also used four impact speeds: 2, 4, 8, and 16 m/s.

**Figure 14:**
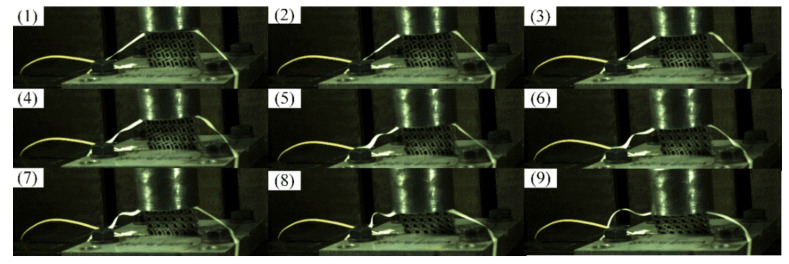
The deformation of the chiral metamaterial during the impact process. ((**1**–**9**) represented the impact compression views which were taken every 300 consecutive photos).

The diagram in Figure 14 shows the deformation of the chiral metamaterial during the impact process captured by the high-speed camera. The high-speed camera had a fixed-point position monitoring function. In conjunction with the displacement sensor in the impact head, it monitored the change in displacement of a specific area of the chiral metamaterial during the impact compression process in real time. It can be seen from the deformation process in Figure 14 that during the impact, the chiral metamaterial had obvious rotational deformation characteristics. Chiral metamaterials disperse and transfer impact loads through the rotation, traction, and deformation of ligaments and cylinders to realize the buffering and damping effect of the overall structure. If we compare the chiral mechanical metamaterial designed in this research with the research results of Su et al. [47], two conclusions can be drawn. Firstly, they found the same reverse slip state at 2–14 m/s rather than a concave deformation state above 28 m/s. Secondly, the chiral mechanical metamaterial designed here still had a uniform structure overall under 25% deformation, avoiding the defects of local aggregation and deformation. These conclusions show that the chiral metamaterial designed here not only has a complete and effective deformation mechanism but also that the energy absorption properties for the overall structure are superior.

**Figure 15:**
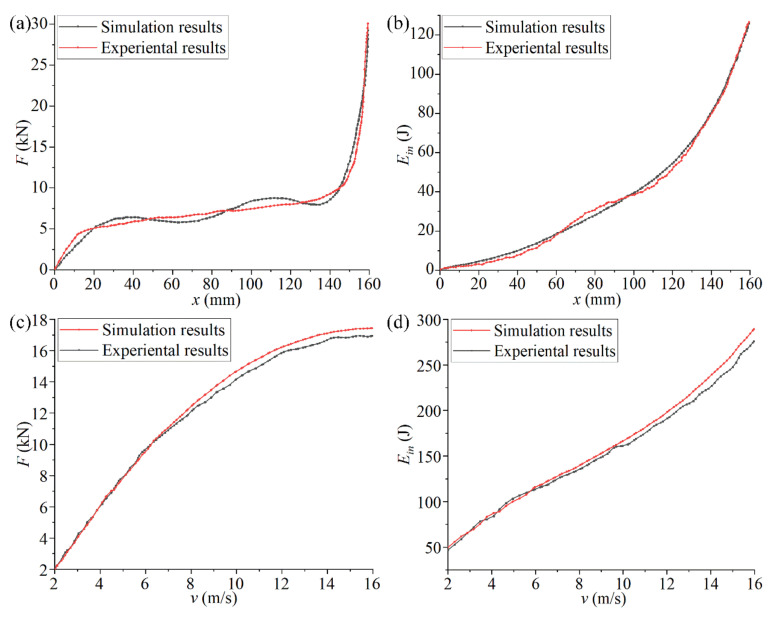
Comparative analysis of the experiment and simulation results for optimized structural. ((**a**,**b**) compare the experimental and simulation results of the optimized structure at the same impact speed (4 m/s). (**c**,**d**) compare the experimental and simulated optimized structure at different speeds from 2–16 m/s).

The diagrams in Figure 15a,b compare the experimental and simulation results of the optimized structure at the same impact speed (4 m/s). In Figure 15a,b, it is observed that the experimental and simulation curves have the same changing trend, but the deviation of the data in the front and the middle sections is more obvious. In this period, the deformation of chiral structures is relatively severe, but the sensors are all arranged on the surface of the structure (unlike the data extraction method used in the simulation analysis), and it is difficult to collect instantaneous change data. Therefore, the curves of the experiment and simulation will have a certain deviation. When the chiral structure entered the densification stage, the increase in the amplitude of the energy absorption curve was obviously smaller than the increasing amplitude of the compression reaction force. This difference shows that the energy generated by the external force is dissipated outside the system in other forms rather than being fully and effectively absorbed by the chiral structure when it entered the densification stage. Therefore, the chiral structure failed in the densification stage, which should be avoided in actual applications processes.

The diagrams in Figure 15c,d compare the experimental and simulated optimized structure at different speeds from 2–16 m/s. In Figure 15c,d, it can be observed that the experimental and simulation curves have the same changing trend, but the fit was higher at low speeds, and the gap gradually increased when the impact speed increased. This is because the system energy was externally dissipated during the experiment. With an increase in the impact speed, the structural rotation and deformation were not timely, which led to the continuous increase in the dissipated energy. This phenomenon cannot be reflected in the simulation, so there will be deviations between the simulation and the experiment.

### 4.3. Feedback Adjustment

On the premise of ensuring that the experimental procedure is complete and effective, we should adjust and optimize the simulation results when there are differences between the experimental and simulation results. Through data analysis of the impact experiment described above, we found that there were certain deviations between the results of the simulation and the experiment. Although the objective reasons in the experiment (such as equipment accuracy, experimental plan, etc.) are analyzed above, we designed a feedback adjustment method, also known as secondary modeling optimization, to ensure the effectiveness of the FIP design.

In the change curves of the structural parameters and output parameters in Figure 7, it can be seen that *L2*, *L3*, *L6* and *L7* gradually stabilized when the output variable stabilized (70 steps). The remaining parameters still fluctuated significantly. This phenomenon showed that the four structural parameters of *L2*, *L3*, *L6*, and *L7* are more sensitive to the output variables. It can be used to construct the sample space of the second round of the surrogate model to enhance the target characteristics of the optimized structures. In feedback adjustment, the constraints of the updated optimization of new sample points were reduced, and the optimization kinetic energy was increased because the parametric dimension of the sample points was reduced. Thus, a new state of convergence can be achieved through the surrogate model.

**Figure 16:**
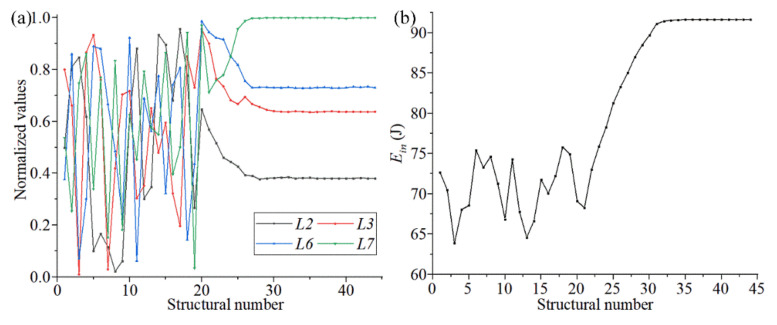
The change trends of the structural parameters (**a**) and output variables (**b**) from the feedback adjustment.

The size parameters of the chiral structure obtained through the feedback adjustment are shown in Table 5. The change trends of the structural parameters and the output variables from the feedback adjustment are shown in Figure 16. Compared with Figure 7, the results for optimization of the size parameters and output variables (absorbed energy) have some changes, but the magnitude of the changes is not large. These results show that the second optimization is only a fine-tuning of the structural parameters and performance, and the main work of parametric optimization still takes place during the first round. Therefore, we also selected some parameters for a third parametric optimization round, and the results showed almost no change.

**Figure 17:**
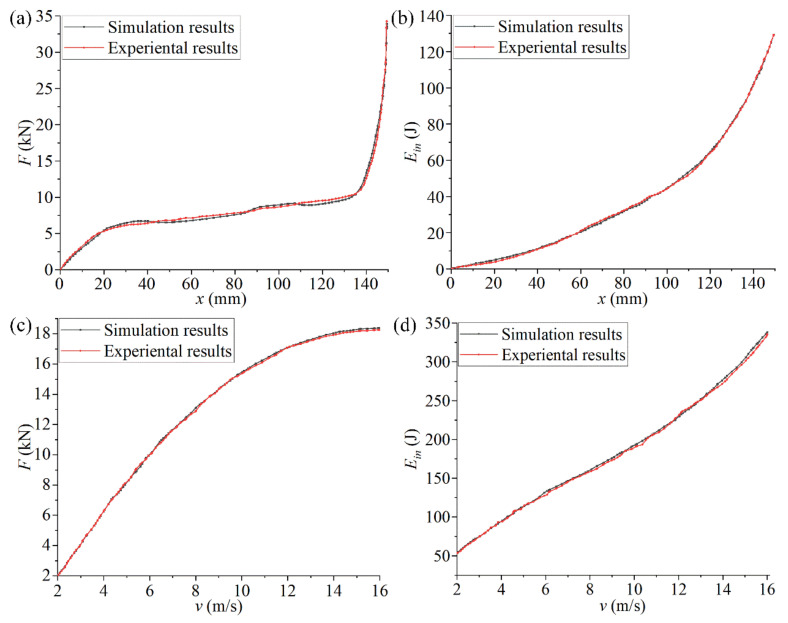
A comparison of the curves of experimental and simulation results for the optimized structure in the feedback adjustment. ((**a**,**b**) show the *x*-*F* curve and the *x*-*E_in_* curve under the same impact velocity. (**c**,**d**) show the *v*-*F* curve and the *v*-*E_in_* curve under different impact velocities).

In order to verify whether the simulation and experimental analysis results of the chiral metamaterials after the feedback adjustment were consistent under the same conditions, an experimental analysis of the compression reaction force *F* and the absorbed energy *E_in_* under the same impact velocity and different impact velocities was conducted with the same setup as the previous impact experiment. Comparisons of the experimental and simulation curves of the optimized structure are shown in Figure 17 to illustrate the effectiveness of the feedback adjustment. Figure 17a,b and show the *x*-*F* curve and the *x*-*E_in_* curve under the same impact velocity. Figure 17c,d show the *v*-*F* curve and the *v*-*E_in_* curve under different impact velocities. 

It can be seen in Figure 17 that the chiral metamaterials optimized by the secondary parameters had a high degree of consistency with the mechanical characteristic curves obtained through experiments and simulations under different conditions. Compared with Figure 15, the deviation in the data of the key stage was greatly reduced. In addition, the new configuration (the new combination of structural parameters) obtained by the feedback adjustment had, to a certain extent, improved the mechanical properties compared with the chiral metamaterial obtained by the first parametric optimization. For example, the compression reaction force *F* and the ability of structural deformation absorption and consumption *E_in_* during the impact process were slightly improved. These changes in the mechanical curves shown in Figure 17 illustrate that the feedback adjustment is of great significance for improving the structural performance and ensuring the effectiveness of parametric optimization. It is also an indispensable link in the FIP design.

## 5. Conclusions

In this study, an FIP design to improve the energy absorption properties of mechanical metamaterials was explored, and a chiral mechanical metamaterial with good energy absorption and impact resistance was obtained based on the rotation mechanism of negative Poisson’s ratio metamaterials.

The chiral mechanical metamaterial obtained by topology optimization had a stronger deformation range and energy absorption properties compared with the initial structure with the same volume fraction. Subsequently, parametric optimization of the initial structure was carried out through the surrogate model, and the overall energy absorption rate was increased by 63%. The analysis results illustrate the transition-slow-dissipation mechanism of the overall structure, indicating the effective range of impact velocity for the chiral mechanical metamaterials and demonstrating the effectiveness of parametric optimization for improving the mechanical properties of chiral mechanical metamaterials.

The large deviation in the results between the experimental and simulation analysis triggered the feedback adjustment process of the FIP design. Feedback adjustment was performed by adjusting the structural parameters and the energy absorption properties of the chiral mechanical metamaterial, resulting in further improvements. The corresponding experimental and simulated mechanical curves were highly consistent, which verified the design effect.

## Figures and Tables

**Table 1:** The parametric value range of the unit cell.

Type	Structural Parameters							
	*L1*	*L2*	*L3*	*L4*	*L5*	*L6*	*L7*	*α1*
Range (mm/°)	80–100	20–35	5–15	45–60	10–20	10–20	20–40	15–20

**Table 2:** The property parameters of the chiral metamaterials.

Type	Property Parameters				
	Density ρ	Elastic Modulus E	Poisson’s Ratio μ	Yield Stress σ	Shear Modulus G
Value	$7.8\;\times \;10^{3}\mathrm{kg}/m^{3}$	$2.1\;\times \;10^{11}\mathrm{Pa}$	0.3	$2.3\;\times \;10^{9}\mathrm{Pa}$	$6\;\times \;10^{10}\mathrm{Pa}$

**Table 3:** The optimized structural parameters of the chiral metamaterials.

Type	Structural Parameters							
	*L1*	*L2*	*L3*	*L4*	*L5*	*L6*	*L7*	α1
Value (mm/°)	100	23	11	52	10	12	40	16

**Table 4:** Typical sample specimens used for the additive manufacturing.

Type	Structures										
	Initial Structure	Optimized Structures									Optimal Structure
	1	41	46	51	56	61	66	71	76	81	88
Number	A	B	C	D	E	F	G	H	I	J	K

**Table 5:** The size parameters of chiral structure after feedback adjustment.

Type	Structural Parameters			
	*L2*	*L3*	*L6*	*L7*
Ranges (mm/°)	18.4–27.6	8.8–13.2	9.6–14.4	32–40
Results (mm/°)	21.9	11.6	13.1	40

## Data Availability

The data presented in this study are available on request from the corresponding author.
